# Risk factors for intrahepatic and extrahepatic cholangiocarcinoma in the United States: A population-based study in SEER-Medicare

**DOI:** 10.1371/journal.pone.0186643

**Published:** 2017-10-19

**Authors:** Jessica L. Petrick, Baiyu Yang, Sean F. Altekruse, Alison L. Van Dyke, Jill Koshiol, Barry I. Graubard, Katherine A. McGlynn

**Affiliations:** 1 Division of Cancer Epidemiology and Genetics, National Cancer Institute, Bethesda, Maryland, United States of America; 2 Stanford Cancer Institute, Stanford University, Palo Alto, California, United States of America; 3 National Heart, Lung, and Blood Institute, Bethesda, Maryland, United States of America; Chang Gung Memorial Hospital Kaohsiung Branch, TAIWAN

## Abstract

**Objectives:**

Intrahepatic (ICC) and extrahepatic (ECC) cholangiocarcinomas are rare tumors that arise from the epithelial cells of the bile ducts, and the etiology of both cancer types is poorly understood. Thus, we utilized the Surveillance, Epidemiology, and End Results (SEER)-Medicare resource to examine risk factors and novel preexisting medical conditions that may be associated with these cancer types.

**Methods:**

Between 2000 and 2011, 2,092 ICC and 2,981 ECC cases and 323,615 controls were identified using the SEER-Medicare database. Logistic regression was used to calculate adjusted odds ratios (OR) and 95% confidence intervals (CI).

**Results:**

Non-alcoholic fatty liver disease was associated with approximately 3-fold increased risks of ICC (OR = 3.52, 95% CI: 2.87–4.32) and ECC (OR = 2.93, 95% CI: 2.42–3.55). Other metabolic conditions, including obesity and type 2 diabetes, were also associated with increased risks of both cancer types. Smoking was associated with a 46% and 77% increased ICC and ECC risk, respectively. Several autoimmune/inflammatory conditions, including type 1 diabetes and gout, were associated with increased risks of ICC/ECC. As anticipated, viral hepatitis, alcohol-related disorders, and bile duct conditions were associated with both cancer types. However, thyrotoxicosis and hemochromatosis were associated with an increased risk of ICC but not ECC, but did not remain significantly associated after Bonferroni correction.

**Conclusions:**

In this study, risk factors for ICC and ECC were similar, with the exceptions of thyrotoxicosis and hemochromatosis. Notably, metabolic conditions were associated with both cancer types. As metabolic conditions are increasing in prevalence, these could be increasingly important risk factors for both types of cholangiocarcinoma.

## Introduction

Cholangiocarcinomas are tumors that arise from the epithelial cells of the bile duct. In low cholangiocarcinoma incidence areas, such as the United States (US), risk factors are not well defined, and it is unclear if cholangiocarcinoma has similar etiology regardless of location within the bile duct–intrahepatic (International Classification of Diseases for Oncology [ICD-O] [1] C22.1) or extrahepatic (ICD-O C24.0). Intrahepatic cholangiocarcinomas (ICCs) are considered by the ICD-O to be liver tumors (ICD-O C22) because they arise in the part of the bile duct that is inside the liver. In contrast, extrahepatic cholangiocarcinomas (ECCs) are classified as biliary tract tumors (ICD-O C24) because they arise in the part of the bile duct that lies outside the liver [1].

Both tumors are associated with preexisting medical conditions, including Caroli disease, primary sclerosing cholangitis, and inflammatory bowel disease [2]. Liver flukes are also a known risk factor for both tumors in some high-risk countries, such as Thailand [2]. While these tumors share many similar risk factors, differences in risk factors have also been reported. Hepatitis C virus (HCV) [3–7], obesity [6], chronic non-alcoholic liver disease [6], and tobacco [6] have been shown to be associated with ICC but not ECC. In the US, incidence rates of ICC have been increasing [8], even when accounting for misclassification of hilar cholangiocarcinoma (i.e., Klatskin tumors) as ICC instead of ECC [9]. While there is some indication that rates of ECC have also been increasing [10], the trend had not been as rapid as ICC [8].

A previous study utilizing the Surveillance, Epidemiology, and End Results (SEER)-Medicare resource, which provides information on medical conditions and co-morbidities recorded in inpatient and outpatient encounters, provided the first information on risk factors for cholangiocarcinoma by anatomic subsite between 1993 and 1999 [6]. As a follow-up to this previous evaluation, we examined risk factors for ICC and ECC, using SEER 18 registries between 2000 and 2011. This study extends prior evaluations by utilizing an expanded SEER registry–providing broader population coverage–and examining novel preexisting medical conditions.

## Materials and methods

### Data source

The National Cancer Institute’s SEER 18 database includes 18 registries (Alaska Native Tumor Registry, Atlanta, Greater California, Connecticut, Detroit, Greater Georgia, Rural Georgia, Hawaii, Iowa, Kentucky, Los Angeles, Louisiana, New Jersey, New Mexico, San Francisco-Oakland, San Jose-Monterey, Seattle-Puget Sound, and Utah) and covers approximately 28% of the US population [11]. For this study, we excluded the Alaska Native Tumor Registry, due to limited sample size.

The US Medicare program is a governmentally administered national health insurance program, which provides coverage for approximately 97% of individuals 65 years and older and also covers individuals with end stage renal disease or disability [12]. Almost all Medicare beneficiaries are enrolled in Part A, which includes hospital, skilled-nursing facility, hospice and some home health care. Of the beneficiaries enrolled in Part A, 96% pay a monthly premium to enroll in Part B, which covers physician and outpatient services [12].

The SEER-Medicare database links approximately 93% of Medicare-aged individuals in SEER cancer registries with claims files from Medicare enrollment [13]. SEER-Medicare is considered exempt research involving human subjects by the National Institutes of Health Office of Human Subjects Research.

### Study population

All Medicare beneficiaries 65 years of age and older who were diagnosed with ICC or ECC between 2000 and 2011 in the SEER 18 registries were eligible for this study. ICCs were identified by using the ICD-O-3 topography code C22.0 (liver) and morphology codes 8160 and 8161 or topography code C22.1 (intrahepatic bile duct) and morphology codes 8032, 8033, 8070, 8071, 8140, 8141, 8160, 8161, 8260, 8480, 8481, 8490, and 8560. ECCs were identified by topography code C24.0 (extrahepatic bile duct) and morphology codes 8032, 8033, 8070, 8071, 8140, 8141, 8160, 8161, 8162, 8260, 8480, 8481, 8490, and 8560 [14].

Between 2000 and 2011, there were 4,988 ICC and 6,307 ECC cases diagnosed. Exclusion criteria included (1) non-malignant behavior (<11 ICC, 11 ECC); (2) missing diagnosis date (27 ICC, 19 ECC); (3) not enrolled continuously in Medicare Parts A and B for a minimum of three years prior to cancer diagnosis, which was necessary to ensure recording of prior medical diagnoses and resulted in a minimum age of 68 years for study participants (1,301 ICC, 1,391 ECC); (4) age younger than 68 at diagnosis (275 ICC, 236 ECC); (5) enrollment in Medicare due to end stage renal disease or disability (<11 ICC, <11 ECC); (6) unspecified diagnostic confirmation of ICC or ECC (306 ICC, 252 ECC); (7) tumor diagnosis solely on autopsy or death certificate (17 ICC, <11 ECC); (8) enrollment in a health maintenance organization (HMO) during the study period, as Medicare HMOs are not required to submit individual claims (909 ICC, 1,321 ECC); (9) prior diagnosis of stomach, colon, lung, pancreas, breast or rectal cancer within 5 years of ICC or ECC diagnosis (57 ICC, 87 ECC). This resulted in 2,092 ICCs (41.9% of diagnosed cases) and 2,981 ECCs (47.3%) that were eligible for this study.

A 5% random sample of Medicare-enrolled beneficiaries residing in the SEER 18 geographic regions and without prior cancer diagnoses were selected as controls. Controls were assigned a “pseudo-diagnosis” date using a random number generator and were subjected to the same inclusion/exclusion criteria as cases. Of the 778,865 eligible controls, exclusions included (1) died before age 68 (32,560); (2) age younger than 68 on December 31, 2011 (187,497); (3) not enrolled continuously in Medicare Parts A and B for a minimum of three years prior to “pseudo-diagnosis” (69,363); (4) enrollment in Medicare due to end stage renal disease or disability (12,393); (5) enrollment in a health maintenance organization (HMO) during the study period (146,472); (6) did not live in SEER registry area by “pseudo-diagnosis” (6,965). This resulted in 323,615 controls that were selected for this study.

### Risk factor assessment

Selected medical conditions and risk factors were identified on the basis of Medicare Part A or B claims for the three years preceding diagnosis date of ICC or ECC (cases) or “pseudo-diagnosis” date (controls). International Classification of Diseases, ninth edition (ICD-9) codes were used to identify selected medical conditions and risk factors (Table 1). As Medicare captures limited information on alcohol use, we instead examined medical conditions known to be related to alcohol use, such as cirrhosis [15]. Similar to a previous study [16], smoking was captured by ICD-9 codes for “personal history of tobacco use,” “tobacco use disorder,” and “toxic effect of tobacco.”

**Table 1:** International Classification of Diseases, ninth edition codes for preexisting medical conditions and risk factors for cholangiocarcinoma.

**Metabolic conditions**	**ICD-9 Codes**
Non-alcoholic fatty liver disease	571.8
Overweight or obesity	278.0, 278.00, 278.01, 278.02, 278.1, V77.8, 783.1
Obesity	278.00, 278.01
Dyslipidemia	272.0, 272.1, 272.2, 272.4, 272.5, 272.7, 272.9
Hypertension	401, 402, 403, 404
Type 2 diabetes/impaired fasting glucose	250.00, 250.02, 250.10, 250.12, 250.20, 250.22, 250.30, 250.32, 250.40, 250.42, 250.50, 250.52, 250.60, 250.62, 250.70, 250.72, 250.80, 250.82, 250.90, 250.92, 790.2
**Viral hepatitis**	
Hepatitis B virus infection	070.2, 070.3, 070.42, 070.52, V0261
Hepatitis C virus infection	070.41, 070.44, 070.51, 070.54, 070.7, V02.62
Unspecified viral hepatitis	070.49, 070.59, 070.9
**Bile duct conditions**	
Caroli disease	751.61
Biliary cirrhosis	571.6
Cholangitis	576.1
Cholecystectomy	51.2, 576.0
Choledochal cysts	751.69
Cholelithiasis	574.00, 574.01, 574.10, 574.11, 574.20, 574.21
Choledocholithiasis	574.30, 574.31, 574.40, 574.41, 574.50, 574.51
**Autoimmune/inflammatory conditions**	
Autoimmune hepatitis	571.42
Rheumatoid arthritis	714.0, 714.2, 714.30, 714.31, 714.32, 714.33
Reactive arthritis	099.3, 711.10, 711.11, 711.12, 711.13, 711.14, 711.15, 711.16, 711.17, 711.18, 711.19
Celiac disease	579.0
Type 1 diabetes	250.01, 250.03, 250.11, 250.13, 250.21, 250.23, 250.31, 250.33, 250.41, 250.43, 250.51, 250.53, 250.61, 250.63, 250.71, 250.73, 250.81, 250.83, 250.91, 250.93
Crohn’s disease	555.0, 555.1, 555.2, 555.9
Ulcerative colitis	556.0, 556.1, 556.2, 556.3, 556.4, 556.5, 556.6, 556.8, 556.9
Chronic pancreatitis	577.1
Gout	274.00, 274.01, 274.02, 274.03, 274.10, 274.19, 274.81, 274.82, 274.89, 274.9
Lupus	710.0
Thyrotoxicosis	242.40, 242.41, 242.80, 242.81, 242.90, 242.91
**Miscellaneous conditions**	
Alcohol-related disorders	571.0, 571.1, 571.2, 571.3, 571.5, 571.6, 303, 305.0, V11.3, V79.1, 291
Smoking	V15.82, 305.1, 989.84
Nonspecific cirrhosis	571.5, 571.6
Human immunodeficiency virus	V08, 042, 043, 044
Duodenal/gastric ulcers	531, 532, 533
Hemochromatosis	275.0

### Statistical analysis

Demographic features and pre-existing medical conditions were compared between cases and controls using *t* tests for continuous variables and χ^2^ or Fisher's exact tests for categorical variables. Logistic regression was used to calculate odds ratios (ORs) and 95% confidence intervals (95% CI). Covariates were determined *a priori*. All models were adjusted for age, race/ethnicity (non-Hispanic white, non-Hispanic black, Hispanic, Asian, or other), geographic region (SEER-18 excluding Alaska registries), and state buy-in status (proxy of lower socioeconomic status, indicating whether a third-party paid a beneficiary's Medicare premium). The case-only odds ratio, or ratio of the odds ratios (ROR), was calculated to assess etiologic heterogeneity. The ROR equals 1 if there is etiologic homogeneity between ICC and ECC [17]. Wald χ^2^ tests were used to determine the significance of variables in the logistic regression models. All p-values are two-sided. To account for multiple comparisons, we also calculated a Bonferroni corrected p-value for 99 comparisons. All analyses were conducted using SAS version 9.3 (SAS Institute, Cary, NC).

## Results

Demographic characteristics of the study population are shown in Table 2. The mean age of both ICC and ECC cases was older than the controls (76.5 years), and ECC cases (79.2 years) were older than ICC cases (78.0 years). ICC cases and controls were more likely to be female (53.4% and 61.7%, respectively), while ECC cases were more likely to be male (51.2%). While the majority of all participants were white (>80% for ICC, ECC, and controls), there were differences in the distributions of race/ethnicity. Geographic location also differed between the groups, but the largest proportions of cases and controls came from the Greater California and New Jersey cancer registries. There was no difference in dual enrollment status between groups.

**Table 2:** Demographic characteristics of the study participants, SEER-Medicare 2000–2011.

	ICC Cases (n = 2,092)		ICC vs controls, P value	ECC Cases (n = 2,981)		ECC vs controls, P value	Controls (n = 323,615)		ICC vs ECC, P value
	n	%		n	%		n	%	
**Mean age, y (SD)**	78.0	6.5	<0.0001	79.2	6.8	<0.0001	76.5	7.7	<0.0001
**Sex**			<0.0001			<0.0001			0.001
Female	1,118	53.4		1,455	48.8		199,583	61.7	
Male	974	46.6		1,526	51.2		124,032	38.3	
**Race/ethnicity**			<0.0001			<0.0001			0.2
White	1,729	82.7		2,411	80.9		270,885	83.7	
Black	111	5.3		193	6.5		25,458	7.9	
Hispanic	47	2.3		82	2.8		7,177	2.2	
Asian	117	5.6		190	6.4		11,177	3.5	
Other/Missing^1^	88	4.2		105	3.5		8,918	2.8	
**Geographic location**			<0.0001			<0.0001			0.006
San Francisco	110	5.3		149	5.0		10,954	3.4	
Connecticut	150	7.2		204	6.8		18,821	5.8	
Detroit	113	5.4		203	6.8		20,701	6.4	
Hawaii	53	2.5		49	1.6		4,377	1.4	
Iowa	163	7.8		203	6.8		19,432	6.0	
New Mexico	49	2.3		58	2.0		9,038	2.8	
Seattle	144	6.9		132	4.4		16,522	5.1	
Utah	36	1.7		54	1.8		8,099	2.5	
Atlanta	59	2.8		76	2.6		9,116	2.8	
San Jose	43	2.1		76	2.6		6,992	2.2	
Los Angeles	196	9.4		280	9.4		23,271	7.2	
Greater California	317	15.2		528	17.7		56,730	17.5	
Kentucky	131	6.3		197	6.6		24,867	7.7	
Louisiana	86	4.1		142	4.8		20,005	6.2	
New Jersey	311	14.9		438	14.7		44,178	13.7	
Greater/Rural Georgia^1^	131	6.3		192	6.4		30,512	9.4	
**State Buy-in Status**^2^			0.3			0.5			0.2
Ever	398	19.0		609	20.4		64,663	20.0	

^1^Combined due to limited sample size.

^2^State buy-in status is a proxy of lower socioeconomic status and indicates that a third-party (i.e., state of residence) paid a beneficiary's Medicare Part B premium.

Distributions of preexisting medical conditions are shown in Table 3. Overall, hypertension, dyslipidemia, and type 2 diabetes were the most common conditions and were more prevalent in both ICC and ECC cases than in controls. Prevalences of non-alcoholic fatty liver disease (NAFLD), obesity, HCV, lupus, alcohol-related disorders, nonspecific cirrhosis, and hemochromatosis were higher in ICC cases compared to ECC cases, while the prevalences of cholangitis, chronic pancreatitis, choledochal cysts, cholelithiasis, and choledocholithiasis were higher in ECC cases than ICC cases.

**Table 3:** Comparison of preexisting medical conditions among ICC cases, ECC cases, and controls, SEER-Medicare 2000–2011^1^.

	Controls (n = 323,615)		ICC Cases (n = 2,092)		ICC vs controls, P value	ECC Cases (n = 2,981)		ECC vs controls, P value	ICC vs ECC, P value
	n	%	n	%		n	%		
**Metabolic conditions**									
Non-alcoholic fatty liver disease	5,053	1.6	100	4.8	<0.0001	113	3.8	<0.0001	0.08
Overweight or obesity	29,905	9.2	209	10.0	0.2	248	8.3	0.8	0.04
Obesity	23,848	7.4	179	8.6	0.04	202	6.8	0.2	0.02
Dyslipidemia	217,755	67.3	1,535	73.4	<0.0001	2,215	74.3	<0.0001	0.5
Hypertension	249,073	77.0	1,727	82.6	<0.0001	2,494	83.7	<0.0001	0.3
Type 2 diabetes/impaired fasting glucose	117,665	36.4	958	45.8	<0.0001	1,323	44.4	<0.0001	0.3
**Viral hepatitis**									
Hepatitis B virus infection	1,200	0.4	25	1.2	<0.0001	31	1.0	<0.0001	0.6
Hepatitis C virus infection	2,161	0.7	58	2.8	<0.0001	57	1.9	<0.0001	0.04
Unspecified viral hepatitis	642	0.2	13	0.6	<0.0001	12	0.4	0.01	0.3
**Bile duct conditions**									
Caroli disease	20	0.01	<11	<0.5	<0.0001	19	0.6	<0.0001	0.06
Biliary cirrhosis	333	0.1	20	1.0	<0.0001	23	0.8	<0.0001	0.5
Cholangitis	647	0.2	93	4.5	<0.0001	244	8.2	<0.0001	<0.0001
Cholecystectomy	221	0.1	<11	<0.5	0.06	<11	<0.4	<0.0001	0.4
Choledochal cysts	462	0.1	48	2.3	<0.0001	121	4.1	<0.0001	0.0006
Cholelithiasis	14,920	4.6	333	15.9	<0.0001	613	20.6	<0.0001	<0.0001
Choledocholithiasis	2,212	0.7	99	4.7	<0.0001	283	9.5	<0.0001	<0.0001
**Autoimmune/inflammatory conditions**									
Autoimmune hepatitis	53	0.02	<11	<0.5	0.3	<11	<0.4	0.4	1.0
Rheumatoid arthritis	16,800	5.2	104	5.0	0.7	174	5.8	0.1	0.2
Reactive arthritis	154	0.05	<11	<0.5	0.3	<11	<0.4	1.0	0.6
Celiac disease	696	0.2	<11	<0.5	0.3	<11	<0.4	0.9	0.5
Type 1 diabetes	29,082	9.0	246	11.8	<0.0001	329	11.0	<0.0001	0.4
Crohn’s disease	1,834	0.6	20	1.0	0.02	27	0.9	0.01	0.9
Ulcerative colitis	3,098	1.0	43	2.1	<0.0001	49	1.6	0.0001	0.3
Chronic pancreatitis	1,237	0.4	21	1.0	<0.0001	74	2.5	<0.0001	0.0001
Gout	17,600	5.4	158	7.6	<0.0001	230	7.7	<0.0001	0.8
Lupus	2,121	0.7	13	0.6	0.8	<11	<0.4	0.005	0.03
Thyrotoxicosis	11,640	3.6	94	4.5	0.03	111	3.7	0.7	0.2
**Miscellaneous conditions**									
Alcohol-related disorders	8,584	2.7	176	8.4	<0.0001	175	5.9	<0.0001	0.0004
Smoking	33,789	10.4	268	12.8	0.0004	427	14.3	<0.0001	0.1
Nonspecific cirrhosis	2,548	0.8	120	5.7	<0.0001	81	2.7	<0.0001	<0.0001
Human immunodeficiency virus	501	0.2	<11	<0.5	0.4	<11	<0.4	1.0	0.7
Duodenal/gastric ulcers	19,192	5.9	176	8.4	<0.0001	273	9.2	<0.0001	0.4
Hemochromatosis	1,514	0.5	20	1.0	0.001	12	0.4	0.6	0.01

^1^Cells with <11 participants are suppressed.

NAFLD was associated with approximately 3-times the risk of ICC (OR = 3.52, 95% CI: 2.87–4.32) and ECC (OR = 2.93, 95% CI: 2.42–3.55) (Table 4). Obesity, dyslipidemia, hypertension, and type 2 diabetes were also associated with increased risks of both cancer types. While overweight or obesity was associated with ICC risk (OR = 1.27, 95% CI: 1.10–1.47), only obesity was associated with ECC risk (OR = 1.17, 95% CI: 1.01–1.35), but this was not significant after Bonferroni correction.

**Table 4:** Adjusted^1^ logistic regression analysis examining the association between selected medical conditions and ICC or ECC, SEER-Medicare 2000–2011.

	ICC Cases (n = 2,092)			ECC Cases (n = 2,981)			ICC vs ECC		
	OR	95% CI	P value	OR	95% CI	P value	ROR	95% CI	P value
**Metabolic conditions**									
Non-alcoholic fatty liver disease	3.52	(2.87–4.32)	**<.0001**	2.93	(2.42–3.55)	**<.0001**	1.20	(0.91–1.59)	0.1924
Overweight or obesity	1.27	(1.10–1.47)	0.0013	1.10	(0.96–1.25)	0.1641	1.16	(0.95–1.41)	0.1464
Obesity	1.42	(1.21–1.66)	**<.0001**	1.17	(1.01–1.35)	0.0334	1.21	(0.98–1.50)	0.0764
Dyslipidemia	1.41	(1.27–1.55)	**<.0001**	1.56	(1.43–1.69)	**<.0001**	0.90	(0.79–1.03)	0.1283
Hypertension	1.39	(1.24–1.56)	**<.0001**	1.43	(1.30–1.58)	**<.0001**	0.97	(0.84–1.13)	0.7065
Type 2 diabetes/impaired fasting glucose	1.54	(1.41–1.68)	**<.0001**	1.45	(1.34–1.56)	**<.0001**	1.07	(0.95–1.19)	0.2766
**Viral hepatitis**									
Hepatitis B virus infection	2.97	(1.97–4.46)	**<.0001**	2.38	(1.65–3.44)	**<.0001**	1.24	(0.72–2.14)	0.4293
Hepatitis C virus infection	4.67	(3.57–6.11)	**<.0001**	3.18	(2.43–4.16)	**<.0001**	1.47	(1.01–2.13)	0.0439
Unspecified viral hepatitis	3.19	(1.83–5.55)	**<.0001**	1.97	(1.11–3.50)	0.0209	1.62	(0.74–3.56)	0.2312
**Bile duct conditions**									
Caroli disease	38.13	(14.20–102.38)	**<.0001**	96.81	(51.02–183.68)	**<.0001**	0.39	(0.15–1.06)	0.0656
Biliary cirrhosis	9.84	(6.24–15.52)	**<.0001**	8.34	(5.44–12.78)	**<.0001**	1.18	(0.65–2.16)	0.5888
Cholangitis	21.52	(17.21–26.90)	**<.0001**	40.80	(34.96–47.60)	**<.0001**	0.53	(0.41–0.68)	**<.0001**
Cholecystectomy	2.74	(1.02–7.38)	0.0464	4.71	(2.49–8.91)	**<.0001**	0.58	(0.18–1.86)	0.3606
Choledochal cysts	15.66	(11.58–21.18)	**<.0001**	27.12	(22.06–33.34)	**<.0001**	0.58	(0.41–0.81)	0.0016
Cholelithiasis	3.93	(3.49–4.43)	**<.0001**	5.29	(4.83–5.80)	**<.0001**	0.74	(0.64–0.86)	**<.0001**
Choledocholithiasis	6.94	(5.64–8.54)	**<.0001**	14.22	(12.48–16.20)	**<.0001**	0.49	(0.39–0.62)	**<.0001**
**Autoimmune/inflammatory conditions**									
Autoimmune hepatitis	3.28	(0.45–23.83)	0.2399	2.49	(0.34–18.08)	0.3677	1.32	(0.08–21.13)	0.8448
Rheumatoid arthritis	0.96	(0.79–1.17)	0.6910	1.10	(0.94–1.28)	0.2353	0.87	(0.68–1.12)	0.2949
Reactive arthritis	2.12	(0.52–8.56)	0.2933	0.74	(0.10–5.32)	0.7670	2.85	(0.26–31.47)	0.3929
Celiac disease	0.44	(0.11–1.78)	0.2503	1.00	(0.45–2.24)	0.9977	0.44	(0.09–2.19)	0.3180
Type 1 diabetes	1.43	(1.25–1.63)	**<.0001**	1.30	(1.16–1.46)	**<.0001**	1.10	(0.92–1.31)	0.2925
Crohn’s disease	1.77	(1.13–2.75)	0.0120	1.71	(1.17–2.51)	0.0058	1.03	(0.58–1.84)	0.9201
Ulcerative colitis	2.18	(1.61–2.95)	**<.0001**	1.75	(1.32–2.33)	**0.0001**	1.24	(0.82–1.88)	0.3073
Chronic pancreatitis	2.66	(1.72–4.10)	**<.0001**	6.61	(5.21–8.40)	**<.0001**	0.40	(0.25–0.65)	**0.0003**
Gout	1.40	(1.19–1.65)	**<.0001**	1.43	(1.25–1.64)	**<.0001**	0.98	(0.79–1.21)	0.8505
Lupus	1.03	(0.60–1.79)	0.9093	0.40	(0.19–0.83)	0.0145	2.61	(1.04–6.55)	0.0412
Thyrotoxicosis	1.25	(1.01–1.54)	0.0367	0.99	(0.81–1.19)	0.8811	1.27	(0.96–1.68)	0.0993
**Miscellaneous conditions**									
Alcohol-related disorders	3.72	(3.17–4.35)	**<.0001**	2.60	(2.23–3.04)	**<.0001**	1.43	(1.15–1.78)	0.0014
Smoking	1.46	(1.28–1.66)	**<.0001**	1.77	(1.59–1.96)	**<.0001**	0.83	(0.70–0.98)	0.0252
Nonspecific cirrhosis	8.26	(6.83–9.99)	**<.0001**	3.83	(3.05–4.80)	**<.0001**	2.16	(1.62–2.88)	**<.0001**
Human immunodeficiency virus	0.36	(0.05–2.55)	0.3054	1.03	(0.38–2.75)	0.9612	0.35	(0.04–3.13)	0.3475
Duodenal/gastric ulcers	1.42	(1.21–1.66)	**<.0001**	1.46	(1.29–1.66)	**<.0001**	0.97	(0.79–1.19)	0.7672
Hemochromatosis	2.07	(1.33–3.22)	0.0014	0.87	(0.49–1.54)	0.6344	2.37	(1.16–4.86)	0.0185

^1^Adjusted for age, gender, race, geographic location, and state buy-in status.

**Bold p-values**: Indicates p-values that remain significant at the Bonferroni threshold.

All viral hepatitis infections (HBV, HCV, and unspecified) and bile duct conditions examined were associated with both ICC and ECC (Table 4). Caroli disease was the strongest risk factor for both cancer types, conferring a 38-fold higher risk of ICC (OR = 38.13, 95% CI: 14.20–102.38) and a 97-fold higher risk of ECC (OR = 96.81, 95% CI: 51.02–183.68), but sample size was limited (i.e., ICC with Caroli disease diagnosis n<11, ECC n = 19).

Several autoimmune/inflammatory conditions, including type 1 diabetes, Crohn’s disease, ulcerative colitis, chronic pancreatitis, and gout were associated with increased risks of both cancer types (Table 4). However, lupus was associated with a decreased risk of ECC (n<11, OR = 0.40, 95% CI: 0.19–0.83) but had no association with ICC (n = 13). Thyrotoxicosis was associated with an increased risk of ICC (OR = 1.25, 95% CI: 1.01–1.54) but not ECC. However, these associations were no longer significant after Bonferroni correction.

Alcohol-related disorders were associated with a 3.7-fold higher risk of ICC (OR = 3.72, 95% CI: 3.17–4.35) and a 2.6-fold higher risk of ECC (OR = 2.60, 95% CI: 2.23–3.04). Smoking was associated with a 46% increased ICC risk (OR = 1.46, 95% CI: 1.28–1.66) and a 77% increased ECC risk (OR = 1.77, 95% CI: 1.59–1.96). Nonspecific cirrhosis and duodenal/gastric ulcers were significantly associated with an increased risk of both cancer types. Hemochromatosis was only associated with an increased risk of ICC (OR = 2.07, 95% CI: 1.33–3.22), but this was not significant after Bonferroni correction.

## Discussion

In this large, US population-based study, risk factors for ICC and ECC were similar, with the exceptions of thyrotoxicosis and hemochromatosis. We report several novel risk factors that are related to both ICC and ECC, which were either not captured or not associated in the previous study comparing ICC and ECC [6]. These include metabolic conditions, viral hepatitis, biliary cirrhosis, Caroli disease, type 1 diabetes, ulcerative colitis, chronic pancreatitis, gout, and nonspecific cirrhosis. HCV infection, alcohol-related disorders, and nonspecific cirrhosis had a stronger association with ICC compared to ECC. Cholangitis, choledochal cysts, cholelithiasis, choledocholithiasis, chronic pancreatitis, and smoking had a stronger association with ECC compared to ICC. Thyrotoxicosis and hemochromatosis were associated with an increased risk of ICC but were not associated with ECC. However, the associations with thyrotoxicosis and hemochromatosis were not significant after correction for multiple comparisons.

In the US, incidence rates of ICC have been increasing, even when accounting for misclassification of hilar cholangiocarcinomas (i.e., Klatskin tumors), while incidence rates of ECC have risen more modestly [8–10]. As shown in this study, HCV infection is a stronger risk factor for ICC. The stronger association between HCV and ICC is expected, as chronic HCV infection is known to damage the intrahepatic bile ducts [19]. Additionally, the prevalence of HCV infection is higher in the cohort of individuals born 1945–1965 (also known as “baby boomers”) [18], who are not captured in the current study. The prevalence of overweight/obesity is also rising in the US [20, 21], and overweight/obesity is a stronger risk factor for ICC than ECC. Thus, the rising prevalence of HCV infection and obesity may explain the rapidly rising incidence of ICC but not ECC.

All metabolic conditions examined were associated with increased rates of both ICC and ECC. A meta-analysis has shown that type 2 diabetes is associated with a 97% increased risk of ICC and a 63% increased risk of ECC [22], similar to what we report in the current study. Hypertension and dyslipidemia were previously associated with ICC in a SEER-Medicare study [23], but have not been examined in relation to ECC. Obesity and NAFLD have been reported to be associated with ICC [6, 24], but their associations with ECC have been less well studied [25]. A recent meta-analysis reported a 52% increased risk of cholangiocarcinoma associated with obesity, but did not stratify by location within the bile duct [25]. Obesity may cause chronic, low-grade systemic inflammation through increased levels of TNF-α, IL-6, leptin, free fatty acids, and TLR4 and decreased adiponectin levels [26]. This systemic inflammation is believed to contribute to metabolic dysregulation, including onset of insulin resistance and subsequent diabetes [27], and the development and progression of NAFLD to steatohepatitis, fibrosis, cirrhosis; possibly culminating in development of cholangiocarcinoma [26]. As the prevalence of obesity [20, 21] and NAFLD [28, 29] is increasing, metabolic conditions are going to be increasingly important risk factors for these cancer types.

Viral hepatitis infections, including HBV, HCV, and unspecified, were associated with both types of cholangiocarcinoma. Two meta-analyses have reported associations between HBV [30] and HCV [31] and ICC and ECC. Additionally, HBV DNA and HCV RNA have been detected in cholangiocarcinoma tissue [32, 33], and HCV can cause bile duct injury [19]. However, it should be noted that the HCV prevalence among controls (0.7%) was low compared to the estimated population prevalence (1.0%) [34], which is likely due to the fact that the controls were less likely to be tested for HCV.

Caroli disease is a rare inherited disorder characterized by a cystic dilation of the intrahepatic bile ducts [35]. This is a known risk factor for cholangiocarcinoma but often manifests at younger ages [2]. In the current study, which is a population over 68 years of age, Caroli disease was associated with the strongest associations of any medical condition–a 38-fold increase in ICC risk and a 97-fold increase in ECC risk.

The ICD codes available in the SEER-Medicare database include “biliary cirrhosis” and “cholangitis” and do not allow us to distinguish autoimmune-mediated cholangiopathies involving progressive destruction of the bile ducts (i.e., primary biliary cirrhosis and primary sclerosing cholangitis) [36, 37] versus obstructions of the bile ducts, which may be manifestations of an underlying cancer (i.e., secondary biliary cirrhosis and ascending cholangitis). Cholangitis had a strong association with both types of cholangiocarcinoma, with a 21-fold increased risk of ICC and a 40-fold increased risk of ECC. Cholangitis involves both the intrahepatic and extrahepatic biliary tree, is associated with inflammatory bowel disease, and is an established risk factor for cholangiocarcinoma [2]. While it has been reported that cholangitis frequently co-occurs with inflammatory bowel disease (i.e., Crohn’s disease or ulcerative colitis) [38], in this population only 12% of ICC and 7% of ECC had a diagnosis of both conditions. Primary biliary cirrhosis occurs in the small intrahepatic bile ducts [36], while secondary biliary cirrhosis can involve the large intrahepatic or extrahepatic bile ducts. Biliary cirrhosis has only been reported to be associated with ICC and ECC in a previous SEER-Medicare study [6]. However, primary biliary cirrhosis is known to be associated with an increased risk of hepatocellular carcinoma [36]. Thus, biliary cirrhosis may affect both the bile ducts and the liver parenchyma.

Biliary cysts (i.e., choledochal cysts), stones in the bile duct (i.e., choledocholithiasis) and gallbladder (i.e., cholelithiasis), and removal of the gallbladder (i.e., cholecystectomy) were associated with both types of cholangiocarcinoma, but more strongly associated with ECC. While choledochal cysts are a congenital condition and relatively uncommon in Western countries, gallstones affect 10–15% of the population [39]. A Swedish study also found an increased risk of ICC and ECC among cohorts of individuals diagnosed with gallstones both with and without cholecystectomy [40]. Possible mechanisms for bile duct stones and cholecystectomy are through inflammation and bile exposure. Gallstones are known to cause inflammation to adjacent organs [41], but gallbladder removal can alleviate inflammation associated with gallstones [42]. However, when the gallbladder is removed, the enterohepatic circulation of bile salts becomes continuous [43]. Thus, this could allow for increased systemic accumulation of bile acids, which are carcinogenic [44]. A previous SEER-Medicare study showed that the risk of cholangiocarcinoma rapidly decreased with increasing time between a cholecystectomy Medicare claim and cancer diagnosis, but cholangiocarcinoma risk was still significantly elevated even six or more years after a gallstone diagnosis [45].

Autoimmune/inflammatory conditions associated with an increased risk of both types of cholangiocarcinoma included type 1 diabetes, inflammatory bowel disease (i.e., Crohn’s disease and ulcerative colitis), chronic pancreatitis, and gout. We hypothesized that these conditions may be related to cholangiocarcinoma through common pathways of chronic inflammation and/or microbiome dysbiosis [46]. Lupus was inversely associated with ECC risk. While this is a plausible association, as lupus patients commonly take steroids and other anti-inflammatory medications, numbers were limited with less than eleven ECC cases diagnosed with lupus. Hyperthyroidism (i.e., thyrotoxicosis) was associated with an increased risk of only ICC. Underlying mechanisms for the association with thyrotoxicosis could involve oxidative stress [47, 48] or increased production of sex hormone-binding globulin [49]. However, it is unclear why either mechanism would increase the risk of only ICC.

As expected, alcohol-related disorders and nonspecific cirrhosis were associated with increased risks of ICC and ECC. The association between smoking and ICC [24] and ECC [50] is controversial. However, we found significant associations between smoking and both types of cholangiocarcinoma. As reported in previous studies [51], duodenal/gastric ulcers, which are thought to be a proxy for *Helicobacter pylori* infection, were associated with increased risks of both ICC and ECC. However, this finding should be interpreted cautiously because cholangiocarcinoma patients, ECC patients in particular, often undergo endoscopy for diagnosis [52]. Thus, this association could partially be due to diagnostic bias. Finally, hemochromatosis is a genetic disorder in which increased dietary iron absorption leads to organ deposition of iron, particularly in the liver [53]. Thus, the association with ICC and not ECC is not unexpected.

The current study is the largest study of ICC and ECC to date, but as the study is based on a database linkage there are several limitations. Selection bias is possible, as only 45% of cases were eligible per our exclusion criteria. The most exclusions were for individuals enrolled in HMOs, as Medicare does not include individual claims for HMO enrollees [54]. This study is based on ICD-9 codes; therefore, it was imperative to have individual claims submitted or we would have no exposure information for individuals. Medicare data are collected for billing rather than research purposes; thus, the prevalence of obesity and smoking is almost certainly underestimated. Additionally, we assessed alcohol-related disorders, as alcohol use is not captured in Medicare data. However, these conditions likely serve as a proxy for individuals with extreme alcohol use. This study only included individuals ages 68 years and older and therefore may not be generalizable to individuals diagnosed at younger ages. However, the median age at diagnosis for ICC is 67 years and for ECC is 72 years [8]. Thus, the results are still largely generalizable to the age demographic at highest risk of developing cholangiocarcinoma.

Strengths of the current study included use of SEER 18 registries, which cover approximately 28% of the US population [11], and use of Medicare data to determine preexisting medical conditions. Within the SEER areas, the SEER-Medicare database links approximately 93% of Medicare-aged individuals (≥65 years of age) in SEER cancer registries with claims files from Medicare enrollment [13]. By using Medicare data, instead of self-report, recall bias was potentially avoided for ascertainment of preexisting medical conditions. While there is potential for misclassification of tumor type, cancer cases were identified through SEER cancer registries. To maintain status as a SEER member, registries must maintain rigorous certification standards, including validation of all codes. SEER registries use ICD-O-3 topography and morphology codes to classify cancers; thus, the accuracy of the diagnoses is quite high.

In conclusion, risk factors for ICC and ECC were similar, with the exceptions of thyrotoxicosis and hemochromatosis which only increased the risk of ICC. Notably, metabolic conditions, including NAFLD, obesity, dyslipidemia, hypertension, and type 2 diabetes, were associated with both cancer types. As metabolic conditions are increasing in prevalence, these could be increasingly important risk factors for both types of cholangiocarcinoma.

## Abbreviations

CI: confidence interval
ECC: extrahepatic cholangiocarcinoma
HBV: hepatitis B virus
HCV: hepatitis C virus
ICC: intrahepatic cholangiocarcinoma
ICD-9: International Classification of Diseases, ninth edition
ICD-O: International Classification of Diseases for Oncology
NAFLD: non-alcoholic fatty liver disease
OR: odds ratio
SEER: Surveillance, Epidemiology, and End Results
